# Faster but Not Truer, Part 2: The Regulatory Record, the Economics, and the TRACE-HEOR Reporting Standard

**DOI:** 10.36469/001c.171942

**Published:** 2026-10-15

**Authors:** Onur Baser

**Affiliations:** 1 Graduate School of Public Health City University of New York (CUNY)

**Keywords:** artificial intelligence, large language models, health economics and outcomes research, health technology assessment, regulatory science, reporting standards, real-world evidence

## Abstract

**Background:**

Part 1 of this series found that measured gains from artificial intelligence (AI) in health economics and outcomes research (HEOR) concentrate in tasks with observable ground truth and disappear where the quantity of interest is a counterfactual. What regulators, health technology assessment (HTA) bodies, and sponsors have said about that division has not been assembled in one place.

**Methods:**

We assembled the primary regulatory, HTA, industry, and journal policy record through September 2026, requiring for each claim a named primary source and, where a quantity was reported, its provenance and whether the reporting party had a commercial interest. We downloaded the Food and Drug Administration (FDA) AI-enabled medical device list in full, tabulated published economic evaluations of AI, and set reported industry efficiency gains against disclosed use in submissions. We then evaluated the field against the task-based productivity framework of Nobel laureate Daron Acemoglu and derived a reporting standard from the documented failure modes.

**Results:**

The FDA has authorized 1614 AI-enabled devices, 1230 (76.2%) in radiology, rising from 18 in 2016 to 335 in 2025, and has received more than 500 drug and biologic submissions containing AI components since 2016; its January 2025 credibility assessment guidance remains a draft and excludes discovery and operational uses. Industry sources report time savings of 40% to 97% in regulatory writing and approximately 80% in payer dossier drafting, but every one of these figures comes from a single-arm comparison without a concurrent control and was reported by the company that deployed or sold the tool. AI use was disclosed in 3 of 1391 HTA documents (0.2%) and 0.6% of one journal’s submissions, against surveys reporting use between 25% and 81%. Nineteen economic evaluations of AI have been published against 1614 authorized devices. No regulator or HTA body has been documented accepting or rejecting an AI-derived endpoint as such.

**Conclusions:**

Current disclosure instruments ask whether AI wrote the text. Reviewers need to know whether AI selected the evidence, defined the cohort, derived the endpoint, or generated the code, and whether the recommendation would change without it.

## INTRODUCTION

Part 1 of this series reported a systematic scoping review of 2476 indexed studies on artificial intelligence (AI) in health economics and outcomes research (HEOR) published between 2010 and 2025 and measured the error rate of its own classification against a blinded independent classifier. Two findings carry forward. First, the size of this literature is not known to within a factor of 2: the corpus reduces to approximately 1271 genuinely HEOR-relevant studies (95% confidence interval [CI], 1131-1410), and the fastest growing label, economic evaluation, is the one that could least reliably be assigned (Cohen kappa, 0.144). Second, measured gains concentrate in tasks with an observable answer and are absent where the quantity of interest is a counterfactual.^1–3^

That division sorts the field into two layers. A clerical layer, comprising screening, extraction, code drafting, and dossier assembly, has measurable ground truth and documented gains. An inferential layer, comprising cohort definition, endpoint derivation, confounder selection, and effect estimation, has no supervised target, because no patient is observed under both the treatment and the comparator. The two layers carry different risks and should be governed differently.

This second article asks what the institutional record says about that distinction. Regulators come first, because a reimbursement dossier is a regulated document and because the FDA has written the most developed framework while becoming one of the largest users of these tools. Health technology assessment (HTA) agencies come second, because they will absorb whatever the automation produces. Industry comes third, because the efficiency claims driving adoption originate there and almost none has been independently replicated. We then turn to how these systems fail, to what productivity economics predicts for a field whose central quantity is unobservable, and to what reporting would make an AI-assisted submission auditable.

## METHODS

The review methods, search strategy, classification dictionary, and validation procedure are reported in full in Part 1 and archived in the public replication package.^4^ This article draws on the narrative evidence review conducted alongside that scoping review.

For the regulatory, HTA, industry, and journal policy record, we searched agency, society, and company sources through September 2026 and required a named primary source for every claim. Where a quantity was reported, we recorded its provenance, whether a peer-reviewed study, a conference abstract, a regulatory document, or a company communication, and whether the reporting party had a commercial interest in the result. Conference abstracts and vendor communications are labeled rather than excluded, because in this field they carry information available nowhere else.

We downloaded the FDA AI-enabled medical device list in full and tabulated authorizations by year and specialty in **Supplementary Table S2**. Published economic evaluations of AI in health care appear in **Supplementary Table S1**, industry efficiency claims with their provenance in **Supplementary Table S3**, journal and society policies in **Supplementary Table S4**, and survey estimates of use and proficiency in **Supplementary Table S5**. **Figure 1** places the instruments on a common timeline.

**Figure 1.**
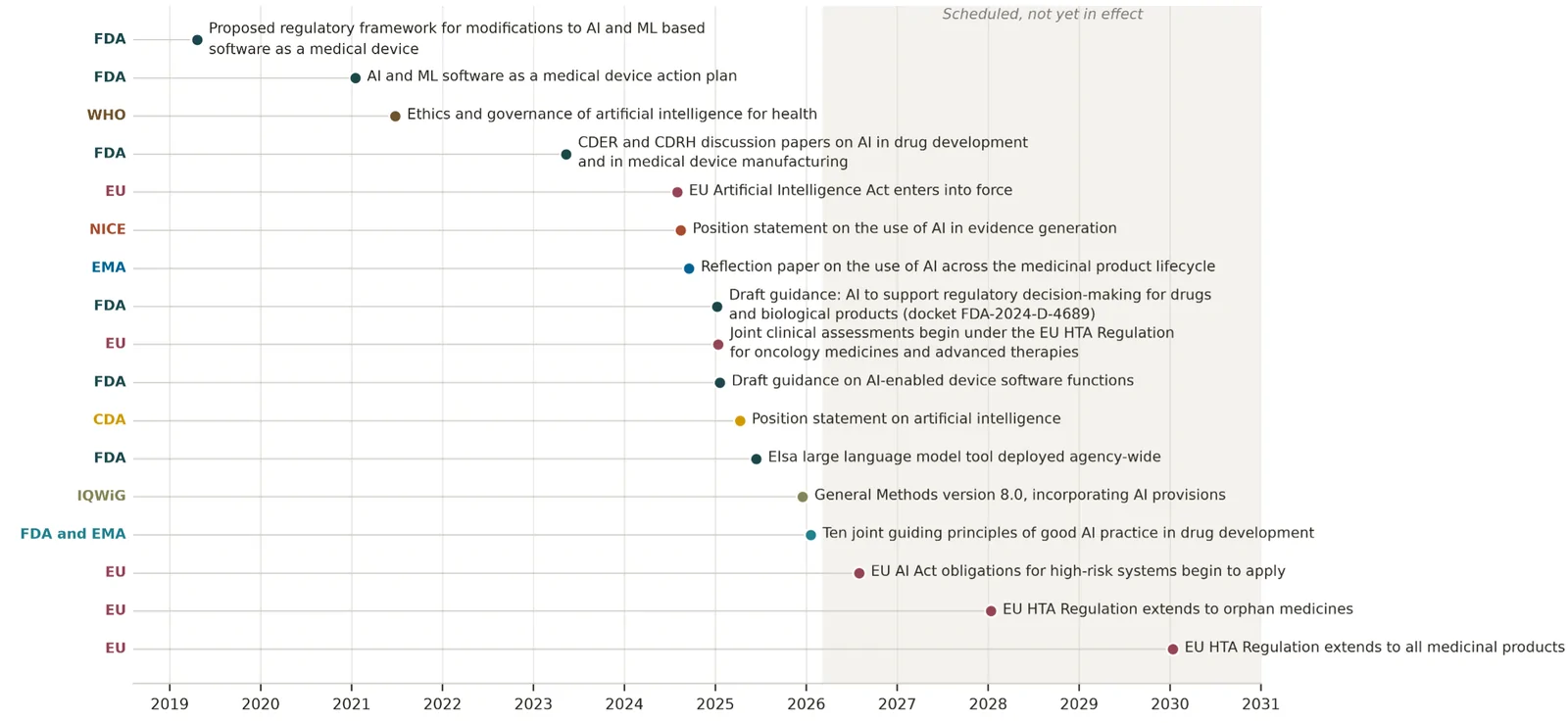
Timeline of Regulatory, HTA, and Journal Policy Instruments Governing AI in Evidence Generation, 2019-2030 Instruments are grouped by issuing domain, with drug and biologic regulation, device regulation, health technology assessment, payer regulation, and publication policy shown separately. Items in force are distinguished from drafts and from provisions with future application dates, including the staged application of Regulation (EU) 2021/2282 from 2025 through 2030 and the general-purpose AI obligations of the EU AI Act from August 2, 2025.

## RESULTS

### The Regulatory Record

The FDA’s position is the most developed and the least finished. **Table 1** summarizes the instruments now in force or in draft.

**Table 1.** Regulatory and HTA Instruments Governing AI in Evidence Generation

**Issuing Body**	**Instrument**	**Date**	**Status**	**Operative Requirement**
US FDA	Considerations for the Use of Artificial Intelligence to Support Regulatory Decision-Making for Drug and Biological Products (docket FDA-2024-D-4689)	Jan 2025	Draft as of Sep 2026; comments closed 7 Apr 2025	Seven step risk-based credibility assessment framework. Model risk is defined as the combination of model influence and decision consequence. Explicitly excludes AI used in drug discovery and in operational efficiencies.
US FDA	Artificial Intelligence-Enabled Device Software Functions: Lifecycle Management and Marketing Submission Recommendations (FDA-2024-D-4488)	Jan 2025	Draft	Recommends performance validation at at least three geographically diverse US clinical sites, with 2-sided 95% confidence intervals reported.
US FDA	AI-Enabled Medical Device List	Current 4 Sep 2026	Published list	1,614 authorizations. Radiology 1,230 (76.2%), cardiovascular 154 (9.5%), neurology 73 (4.5%). Rose from 18 authorizations in 2016 to 335 in 2025.
US FDA	Considerations for the Use of Real-World Data and Real-World Evidence, electronic health records and medical claims (FDA-2020-D-2307)	2024	Final guidance	States that FDA does not endorse any specific AI technology for real-world data curation.
US FDA and EMA	Guiding Principles of Good Artificial Intelligence Practice in Drug Development	Jan 2026	Joint guiding principles	Ten principles including human-centric by design, risk-based approach, clear context of use, data governance and documentation, risk based performance assessment, and life cycle management.
US FDA	Digital Health Advisory Committee on generative AI-enabled devices	20-21 Nov 2024	Advisory committee meeting	First dedicated advisory committee consideration of generative AI in regulated devices.
EMA	Reflection paper on the use of artificial intelligence in the medicinal product lifecycle (EMA/CHMP/CVMP/83833/2023)	9 Sep 2024	Final reflection paper	States that the use of transparent models is preferred. Applies a risk-based approach across the product lifecycle.
EMA CHMP	Qualification opinion on prognostic covariate adjustment (PROCOVA)	2022	Qualification opinion	Qualified for use, while stating that excluding dropout data would not be acceptable for regulatory purposes.
European Union	Regulation (EU) 2024/1689, the AI Act	In force 1 Aug 2024	In force; general purpose AI obligations apply from 2 Aug 2025	Risk tiered obligations. EFPIA has argued that AI used solely for medicines research and development is exempt under Recital 25 and Articles 2.6 and 2.8.
European Union	Regulation (EU) 2021/2282 on health technology assessment	In force 11 Jan 2022	Applies from 12 Jan 2025 for oncology and ATMPs; 13 Jan 2028 for orphans; 13 Jan 2030 for all others in scope	Joint clinical assessment. Approximately 50 assessments of medicines and 5 of devices estimated for 2026, with 12 ongoing and 14 countries involved.
NICE	Use of AI in evidence generation: NICE position statement (ECD11)	15 Aug 2024	Final position statement	AI should only be used when there is demonstrable value from doing so. Where more explainable methods are potentially robust, those should be presented in the first instance. Directs submitters to the PALISADE checklist.
Canada’s Drug Agency (CDA-AMC)	Guidance on Reporting the Use of AI in Evidence Generation	Apr 2025	Final guidance	Built on the principle of augmentation, not replacement. Permits AI generated search strategies, screening, extraction, meta-analysis code, and synthetic or external controls subject to transparent reporting.
IQWiG	General Methods, version 8.0	2025	Final methods manual	German reference methods manual, incorporating requirements applicable to AI derived evidence.
ICER and Peterson Health Technology Institute	Assessment framework for digital health technologies	2024	Published framework	Extends value assessment methods to digital and algorithm-based technologies.
CMS	HPMS memorandum on Medicare Advantage utilization management (CMS-4201-F)	6 Feb 2024	Final rule guidance	Algorithms or AI alone cannot be used as the basis to deny admission.
State of California	Senate Bill 1120, Chapter 879, Statutes of 2024	2024	Enacted statute	Requires licensed physician review of medical necessity determinations rather than reliance on an algorithm alone.
WHO	Ethics and governance of AI for health: guidance on large multimodal models	2024	Guidance	Notes hallucination rates of ≥3% and as high as 27%.
ISPOR	Generative AI taxonomy, ELEVATE-GenAI reporting guideline, and Working Group report for HTA	2024-2025	Published society guidance	First HEOR-specific reporting instruments for generative AI. AI ranked first among HEOR trends for 2026-2027.

Status is as of September 9, 2026. Quoted text is verbatim from the cited instrument. Full citations with uniform resource locators appear in the reference list.

For drugs and biologics, the January 2025 draft guidance proposes a 7-step credibility assessment framework in which a sponsor defines the question of interest, the context of use, and the model risk, then establishes credibility proportionate to that risk.^5^ The definition of model risk is the operative sentence: “Model risk is a combination of two factors: (a) model influence… and (b) decision consequence.” Two features are routinely missed. The guidance explicitly excludes AI used in drug discovery and in operational efficiencies, so most publicly announced pharmaceutical AI deployments fall outside it. It remains a draft as of September 2026, with the comment period having closed on April 7, 2025. The agency’s account of the evidence behind it names an FDA-sponsored workshop convened by the Duke Margolis Institute for Health Policy in December 2022, more than 800 comments from 65 entities, and experience with more than 500 submissions containing AI components since 2016.^6,7^ The Pharmaceutical Research and Manufacturers of America (PhRMA) asked the agency to clarify which device provisions apply, how human in the loop mitigation affects the risk assessment, and how models composed of multiple models are treated,^8^ a fair summary of what the framework does not yet answer.

For devices, the record is quantitative. We downloaded the FDA AI-enabled medical device list in full: 1614 authorizations current as of September 4, 2026, of which 1230 (76.2%) are in radiology, 154 (9.5%) in cardiovascular, and 73 (4.5%) in neurology, rising from 18 authorizations in 2016 to 335 in 2025 and 181 in the first 8 months of 2026.^9^ The January 2025 draft guidance on AI-enabled device software functions asks for validation at “at least three geographically diverse US clinical sites” with 2-sided 95% confidence intervals (CIs).^10,11^ The concentration in radiology is the number a health economist should notice: three-quarters of authorized AI in American medicine operates in one specialty, so the cost-effectiveness literature is being asked to generalize from a narrow deployment base.

The agency is also now a user. Elsa was deployed agency-wide in June 2025 on a FedRAMP High environment, with the explicit statement that it does not train on data submitted by regulated industry.^12^ A regulator that uses these tools internally will develop views about their failure modes faster than one that only reviews them.

The European position is more prescriptive on one dimension. The European Medicines Agency reflection paper states that “the use of transparent models is preferred,”^13^ a preference the National Institute for Health and Care Excellence (NICE) echoes in requiring that where more explainable methods are potentially robust “those should be presented in the first instance, with supplementary use of less transparent approaches.”^14^ The EU AI Act entered into force on August 1, 2024 with general purpose AI obligations applying from August 2, 2025,^15^ and the European Federation of Pharmaceutical Industries and Associations (EFPIA) has argued that AI used solely for medicines research and development is exempt under Recital 25 and Articles 2.6 and 2.8.^16^ Industry association frameworks converge on similar content.^17–19^

Payer-side regulation has moved faster than either. The Centers for Medicare and Medicaid Services stated in February 2024 that “algorithms or artificial intelligence alone cannot be used as the basis to deny admission” in Medicare Advantage,^20^ and California enacted Senate Bill 1120 requiring physician review of medical necessity determinations.^21^ World Health Organization guidance on large multimodal models notes hallucination rates of “at least 3 percent” and “as high as 27 percent.”^22,23^ The asymmetry is worth stating plainly: using AI to deny care is now regulated in US statute, while using AI to generate the evidence that determines whether care is covered at all is governed by voluntary position statements.

### The HTA Record

**Table 2** sets the claims against the record.

**Table 2.** Reported Performance and Efficiency of AI Across HEOR Workflows, with the Provenance of Every Claim

**HEOR Workflow**	**Task**	**Reported Result**	**Comparator**	**Provenance**
Evidence synthesis	Title and abstract screening	Sensitivity 0.962, specificity 0.996; ~110 records/min at US $59	Human reviewer decisions in 1381 and 3146 record sets	Peer-reviewed
Evidence synthesis	Title and abstract screening	Sensitivity 81.1%-96.5%, specificity 25.8%-80.4%; 10,279 false positives in the worst configuration	Human reviewers across 22 665 citations	Peer-reviewed
Evidence synthesis	Title and abstract screening	Sensitivity 0.85, specificity 0.98, with a median specificity gap of 0.48 across reviews	Human reviewers across 16 669 citations	Peer-reviewed
Evidence synthesis	Title and abstract screening	Sensitivity 0.42, kappa 0.34 against human reviewer kappa 0.77	2 human reviewers	Peer-reviewed
Evidence synthesis	Screening prioritization	Work saved over sampling 0.55; ~75% of screening effort saved at recall above 95%	Exhaustive human screening of 11 984 records	Peer-reviewed
Evidence synthesis	Agentic screening and extraction, human in the loop	Screening F1 0.917-0.977, kappa 0.844-0.906; extraction F 0.96-0.998	Human reviewers	Peer-reviewed
Evidence synthesis	Data extraction	96.3% accuracy across 160 extracted items	Human extraction	Peer-reviewed
Evidence synthesis	Data extraction	94.8% accuracy at 88 seconds per randomized trial	Human extraction	Peer-reviewed
Evidence synthesis	Data extraction	Model 96.2%, human 95.3%, human-assisted by model 97.9%; time 86.9-14.7 minutes	Human extraction across 12 814 extractions in 107 trials	Peer-reviewed
Evidence synthesis	Time and cost	17/25 studies reported time reductions above 50%, range 36.0% to more than 99.0%	Unaided human workflow	Peer-reviewed
Evidence synthesis	Time and motion, hybrid vs autonomous	Hybrid 97.0% accuracy, 80.6% recall, 51.4% time saved; autonomous 81.1% time saved but 48.4% recall; unaided humans 90.1% accuracy, 88.7% recall	Unaided human workflow across 234 publications	Conference abstract
Economic modeling	Automated model coding in R	14/15 scripts error-free in the lung cancer set; every error-free script reproduced the published ICER to within 1%	Published cost effectiveness results	Peer-reviewed
Economic modeling	Network meta-analysis input extraction	>99% extraction accuracy	Human extraction	Peer-reviewed
Economic modeling	Expected value of partial perfect information by metamodeling	12.5 million model runs reduced from more than 1000 days to ~2 days, a roughly 500-fold reduction	Brute force nested simulation	Peer-reviewed
Economic modeling	Generative AI in health economic evaluation, evidence base composition	25 studies included from 2322 screened; 92% conference abstracts, 92% published 2024, 96% company authored	Not applicable	Preprint
RWD and causal inference	High-dimensional propensity score	Estimated relative risk moved from 1.09 crude to 0.94 with 15 investigator specified covariates to 0.88 with 500 algorithmically selected covariates	Investigator specified propensity score	Peer-reviewed
RWD and causal inference	High dimensional propensity score weighting, simulation	Bias 0.094 with 23.6% CI coverage, against 0.018 bias and 91.4% coverage for a modified procedure	Known simulated truth	Peer-reviewed
RWD and causal inference	Trial emulation, PARADIGM-HF	Logistic propensity model AUC 0.79 gave HR 0.93; gradient boosted model AUC 0.95 gave HR 0.61	Randomized trial HR 0.81 (95% CI 0.61-1.06)	Preprint
Unstructured data	Social and behavioral determinants from notes	Prevalence recorded rose from 0.03% to 8.3% in claims to 1.40% to 36.3% with notes; lack of social support text only in 99.8% of cases	Structured claims and Z codes	Peer-reviewed
Unstructured data	ECOG performance status extraction	AUROC 0.95 across 79 698 patients and 495 862 notes; imputed poor performance status HR 11.9	Documented performance status	Peer-reviewed
Unstructured data	Oncology variable extraction	Stage value precision 98.8% and recall 92%, but histology linked to neoplasm recall 79%	Human abstraction of 200 patients	Peer-reviewed
Unstructured data	Cancer registry staging extraction	T stage F1 0.89, M stage F1 0.24	Registry abstraction	Peer-reviewed
Unstructured data	Real-world progression-free survival derivation	Sentence-level accuracy 98.2%; patient-level within 30 days 88%; median rwPFS 20 months (95% CI 18-25) computationally vs 25 months (15-35) manually; 30 months from radiology reports vs 23 months from clinical notes	Manual curation in a 100 patient subset	Peer-reviewed
Unstructured data	Human-abstracted real-world response, the reference standard	Inter-abstractor agreement 0.94 (0.92-0.96) on assessment presence and 0.82 (0.78-0.86) on best response; 15.0 minutes per patient line; Spearman 0.99 vs trial ORR	Second human abstractor	Peer-reviewed
Unstructured data	Scaled machine learning real-world response	r = 0.86 against human abstraction in a test subset of 4047	Human abstraction	Conference abstract
Unstructured data	AI-augmented vs traditional real-world data	Recall 49.9%-93.2%, precision 73.6%-93.5%, completeness 46.1% (38.2-54.0) to 96.6% (85.8-107.4)	Traditional structured approach	Peer-reviewed
Unstructured data	Vendor accuracy claim	>92% accuracy	Not stated	Company communication
Synthetic data	Control-arm simulation, generator comparison	CART-based generation recovered the real median PFS inside the 95% CI in 88.8%-98.1% of replicates, against 6.5%-37.8% for a conditional tabular GAN	Real trial control arm	Peer-reviewed
Synthetic data	Synthetic control arm in myelodysplastic syndromes	Clinical fidelity 93%, genomic fidelity 90%, 7133 patients, 187 matched synthetic controls	Real patient records	Peer-reviewed
Synthetic data	Reporting completeness	70/73 studies (95%) evaluated utility; 31 of 67 (46%) claiming privacy purpose evaluated privacy; 8 of 73 (11%) used a differentially private generator	Not applicable	Peer-reviewed
Regulatory writing	Clinical study report first draft	40% faster first draft; manuscript submission timeline 15% shorter	Historical internal process	Company communication
Regulatory writing	Clinical study report first draft	180 hours to 80 hours per fully human-reviewed draft; errors reduced 50%; first draft in as little as 5 minutes	Historical internal process	Company communication
Regulatory writing	Clinical study documentation	>10 weeks to 10 minutes; 90% reduction in CSR writing time; review cycles halved; 95% reduction for device verification protocols	Historical internal process, baseline 2.3 CSRs per writer per year	Company communication
Regulatory writing	Analysis and tabulation process	20-week process projected to be cut approximately in half	Historical internal process	Company communication
Regulatory writing	IND document generation	~100 hours to 3.7 hours for 18 870 pages across 61 documents; to 2.6 hours for 11 425 pages across 58 documents	Historical estimate, no concurrent control, n = 2 INDs	Preprint
Regulatory writing	Industry baseline	~120 hours of regulatory writing over 4 weeks; 83 days from database lock to approved CSR	Not applicable, baseline only	Company communication
Clinical development	Site selection for a 14 000 patient cardiovascular outcomes trial	4-6 weeks reduced to a 2-hour meeting; enrollment closed 13 patients above target	Historical internal process	Company communication
Clinical development	Late-stage study cost	Approximately GBP 8 million saved; all trials targeted 15% faster	Historical internal process	Company communication
Market access	Payer dossier drafting per AMCP Format v5.0	Draft ~90% complete; ~80% less development time; 70%-75% lower cost; authors noted repetition in all sections	Single team, no comparator, 110 source documents	Conference abstract
Market access	Payer policy monitoring	Policy and formulary alerts within 24-48 hours; analysis in minutes rather than days or weeks	Not stated	Company communication
Market access	Pricing, gross to net, contracting, and international reference pricing analytics	No primary source with a measured, attributable efficiency or accuracy figure was located	Not applicable	Not available
Drug discovery	AI-discovered molecule phase success	Phase 1 success 80%-90%; phase 2 success approximately 40%	Historical benchmarks of 13.8% phase 1 to approval overall and 3.4% in oncology	Peer-reviewed
Drug discovery	AI-discovered assets in clinical development	31 assets across 8 companies; 117 assets across 63 companies, with 60 of 117 (51.3%) completing phase 1 and 8 of 117 (6.8%) completing phase 2	Total clinical pipeline of several thousand	Peer-reviewed

Abbreviations: AUC, area under the curve; AUROC, area under the receiver operating characteristic curve; GAN, generative adversarial network; HR, hazard ratio; ICER, incremental cost-effectiveness ratio; ORR, objective response rate; rwPFS, real-world progression-free survival.

Provenance is classified as peer reviewed, preprint, conference abstract, or company communication. Company communication denotes a figure reported by the organization deploying or selling the tool, without independent replication or a concurrent control. Every company reported efficiency figure in this table falls into that category. Where no primary source with a measured figure could be located, the cell records that absence rather than an estimate.

NICE published its position statement on August 15, 2024, requiring demonstrable value, a preference for explainable methods, justification of assumptions using instruments such as PALISADE, and naming algorithmic bias, cybersecurity, and reduced human oversight as the principal risks.^14^ Canada’s Drug Agency followed in April 2025, drawing explicitly on NICE, permitting model generated search strategies, automated screening and extraction, meta-analysis code, and synthetic data and external control arms subject to transparent reporting, and framing the whole underaugmentation rather than replacement.^24–27^

The workload argument for automation is real. Regulation (EU) 2021/2282 applies from January 12, 2025, for new oncology active substances and advanced therapy medicinal products, from January 13, 2028, for orphan products, and from January 13, 2030, for all others in scope.^28–30^ The HTA Coordination Group estimated approximately 50 joint clinical assessments of medicines and approximately 5 of devices for 2026, with 12 ongoing and 14 countries involved as of December 2025.^31,32^ A doubling of dossier volume with no increase in HEOR headcount is the strongest business case for automation available.

Yet, disclosed use remains 3 documents out of 1391, at NICE technology appraisals TA1154 for AI-assisted study screening, TA1071 for AI-assisted searches to identify treatment effect modifiers, and TA962 for a natural-language processing approach, with no explicit reporting in any Canada’s Drug Agency or IQWiG dossier.^33^ A separate audit reported that 7 agencies are adopting AI internally, with NICE recording 49 AI initiatives across 25 appraisals.^34^ Agencies are adopting faster than submitters are disclosing. Conference activity confirms both volume and concentration: of 28 184 abstracts submitted to ISPOR and ISPE between January 2020 and December 2024, 1153 addressed AI, with 89% of applications in literature review automation and 9% in economic modeling.^35–37^

### The Industry Record

For a pharmaceutical reader, the question is not whether these tools work but whether the reported gains are the kind a budget can be built on. **Table 2** and **Supplementary Table S3** present the claims with provenance labeled.

Reported reductions in regulatory writing time span Pfizer at 40% faster first draft clinical study reports^38^; Merck at 180 hours to 80 hours per fully human-reviewed first draft, with errors reduced by 50%^39^; Novo Nordisk at more than 10 weeks to 10 minutes for clinical study documentation^40^; Takeda at a projected halving of a 20-week tabulation process^41^; and a Takeda and Weave Bio preprint at a 97% reduction, from about 100 hours to 3.7 hours for 18 870 pages across 61 source documents in one investigational new drug application.^42^ For comparison, Certara, which sells regulatory writing software, describes the usual baseline: a clinical study report takes about 120 hours of writing over 4 weeks, and a fully reviewed and approved report takes an average of 83 days from database lock. Certara does not, however, report a measured time saving for its own product.^43^

Each of the reductions listed above is reported by the party deploying or selling the tool, and none has been independently replicated. The most methodologically explicit, the Weave and Takeda preprint, compares against a historical estimate with no concurrent control and sample size of 2. Elsewhere, Novartis reported site selection falling from 4 to 6 weeks to a 2-hour meeting for a 14 000-patient trial, and GSK reported approximately £8 million saved in late-stage asthma studies.^44–49^

On the market-access side, the quantified evidence reduces to a single conference abstract. A retrieval augmented generation multi-agent pipeline ingesting 110 documents and generating sections per AMCP Format version 5.0 produced a draft reported as approximately 90% complete with approximately 80% less development time, while the authors noted repetition in all sections and subject-matter experts who only somewhat agreed that responses were largely complete.^50,51^ Vendor claims for payer landscape monitoring are unaudited.^52,53^ For pricing, gross to net, contracting analytics, and international reference pricing, we located no primary source with a measured, attributable efficiency or accuracy figure. That absence is itself a finding.

Against the efficiency claims stand the failures. DSP-1181, presented as the first AI-designed drug when it entered the clinic in January 2020, was abandoned in January 2022 for failing to meet study criteria, and EXS-21546 was discontinued after modeling suggested a suitable therapeutic index would be difficult to reach, with Exscientia, BenevolentAI, and Recursion having accumulated a combined deficit above US $1.5 billion.^54,55^ Independent analyses of AI-discovered molecules report phase 1 success of 80% to 90% and phase 2 success of approximately 40%,^56^ against historical benchmarks of 13.8% overall probability of success from phase 1 to approval and 3.4% in oncology across 406,038 trial entries.^56,57^ Counts of AI discovered candidates in trials range from 31 across 8 companies^58^ to 117 assets across 63 companies,^59^ against a total pipeline of several thousand. Phase 2 is where efficacy is tested, and phase 2 success for AI-discovered molecules sits at the historical average, judged against a cost per approved drug of US $2558 million capitalized preapproval in 2013 dollars^60^ or a median of US $985.3 million (95% CI, 683.6-1228.9) in 2018 dollars.^61–63^

### Failure Modes

Four failure modes are documented well enough to be expected rather than exceptional.

**Bias in deployed algorithms:** Obermeyer et al showed that a commercial risk algorithm applied to approximately 200 million Americans, by using cost as a proxy for need, identified 17.7% of Black patients for extra help where correcting the target would have identified 46.5%.^64^ The mechanism was label choice, not model class, which is why it is a HEOR problem: the outcome variable was a health economics variable.

**Weak external validity**: The Epic sepsis model, evaluated externally across 38 455 hospitalizations, achieved area under the curve of 0.63 (95% CI, 0.62-0.64) against a vendor reported range of 0.76 to 0.83.^65^ High risk of bias is the norm rather than the exception here.^66,67^

**Post deployment decay:** Rahmani et al documented data drift across 112 972 encounters,^68^ and Kopanitsa found across 4 deployed clinical AI systems that calibration degraded earlier and more consistently than discrimination.^69^ For HEOR, this is the crux, because a cost-effectiveness result depends on calibration, not on ranking. A model that still discriminates well and no longer calibrates will keep passing the metric that is monitored and stop supporting the estimate that is used.

**Human factors:** Jabbour et al showed in a randomized vignette study of 457 clinicians that a standard AI raised diagnostic accuracy from a baseline of 73.0% by 2.9 percentage points, while a systematically biased AI lowered it by 11.3 points, with explanations providing no significant protection.^70^ Dratsch et al found experienced radiologists falling from 82.3% to 45.5% accuracy when shown an incorrect AI suggestion,^71–73^ and Budzyń et al reported adenoma detection in non-AI-assisted colonoscopies falling by 6.0 percentage points after AI exposure, from 28.4% to 22.4% (95% CI, 10.5 to -1.6 lower; *P* = .0089).^74,75^ Goh et al randomized 50 physicians and found diagnostic reasoning scores of 76% with a large language model against 74% without, an adjusted difference of 2 percentage points (95% CI, -4 to +8; *P* = .60), while the model alone scored 16 points higher than physicians (95% CI, 2-30; *P* = .03).^76^ The model beat the physician and beat the physician using the model. Any workflow that assumes a human reviewer will catch the model’s errors is assuming away the best measured finding in this literature.

Fabrication is the failure mode most specific to evidence work. Chelli et al assessed 471 references and found hallucination rates of 39.6% for GPT-3.5, 28.6% for GPT-4, and 91.4% for Bard,^77^ and Walters and Wilder examined 636 citations and found 55% fabricated by GPT-3.5 and 18% by GPT-4.^78^ This has already reached consequential documents: a US federal health policy report cited studies that do not appear to exist, with a listed first author confirming she had never written the paper attributed to her,^79^ a filed expert declaration in federal court contained a citation to a nonexistent statistics article,^80,81^ and one journal retracted 129 papers in the first 6 weeks of 2025.^82^ No equivalent incident has yet been documented inside a drug regulatory or HTA submission. That is the state of the record, not a reassurance.

### The Economic Evaluation of AI Itself

HEOR is being asked to evaluate AI and has barely done so. El Arab et al found 19 economic evaluations of AI in health care published from 2020 through 2024, with CHEERS scores between 21 and 27 of 28.^83^ Nineteen evaluations against 1614 authorized AI-enabled devices^9^ is the field’s most direct self-indictment (**Supplementary Table S1**).

The evaluations that exist are instructive. Mital and Nguyen estimated an incremental cost effectiveness ratio of US $23 755 per quality-adjusted life-year for AI vs polygenic risk scores in breast cancer risk stratification, with an AI cost threshold of US $318 per mammogram.^84,85^ Against this, Vargas-Palacios et al modeled AI in the UK breast screening program and found a scenario in which no price per scan made the technology cost effective.^86^ That is the result to hold onto: for a diagnostic AI, cost effectiveness is frequently determined by the downstream care pathway rather than by the price of the algorithm, so a procurement negotiated on price per scan is negotiating the wrong parameter.^87–89^

The regulatory record on external controls is more informative than the synthetic data literature acknowledges. Jahanshahi et al identified 45 FDA approvals between 2000 and 2019 that accepted external control data, 36 (80%) in rare disease,^90,91^ and in its qualification opinion on prognostic covariate adjustment, the EMA Committee for Medicinal Products for Human Use stated that excluding dropout data “would not be acceptable for regulatory purposes.”^92^ The model may be qualified, and the analysis population still has to be complete.

### Transparency, Disclosure, and Workforce

The baseline was poor before AI was introduced. In a random sample of 200 health-economic evaluations, 10 (5%) mentioned access to raw data and analytic code,^93^ and in healthcare discrete event simulation 47 of 564 studies (8.3%) cited an openly available model.^94–97^ Replication attempts are sobering: of 5 economic analyses reimplemented, 2 were fully replicated and 2 were unequivocally not replicable^98^; 4 obesity models rebuilt from 87 publications showed cost-utility ratio deviations from 3.0% lower to 17.9% higher.^99^

Disclosure of AI use runs far below reported use. Journals and societies have converged on rules that prohibit AI authorship and require disclosure of model name, version, and dates.^100–109^ These are tabulated in **Supplementary Table S4**. Against those rules, 0.6% of submissions to *The Lancet Infectious Diseases* in February 2024 declared ChatGPT use and 1.8% declared AI as a research tool,^110^ while surveys report use between 25% and 81%, including more than half of about 1600 academics across 111 countries having used AI in peer review and 81% of 1692 physicians aware of or using AI in 2026 against 38% in 2023.^111–116^ The survey estimates are shown in **Supplementary Table S5**.

Within HEOR, the workforce is not yet equipped. Of 133 HEOR, real-world data, and medical affairs professionals surveyed in April 2026, 61% rated their AI proficiency as basic or very limited and 4% as expert.^117^ Among 100 HEOR leaders at 72 companies, 69% personally use AI, 29% expect AI to replace some HEOR staff within 2 years, and 59% report that their team does not use AI for any listed HEOR workflow.^118^ Medical affairs reports higher confidence than capability, with 49% still using full manual expert review of every AI generated output.^119,120^ Competency frameworks are appearing, but none is HEOR specific,^121–126^ while capital has already moved: AI-enabled startups took 54% of US digital health funding in 2025 against 37% in 2024.^127–131^

## DISCUSSION

The governance finding is the sharpest contrast in this series. Adoption claims run at 40% to 97% time savings in pharmaceutical regulatory writing and approximately 80% in payer dossier drafting, while disclosed use in HTA submissions is 3 of 1391 documents (0.2%) and declared use in journal submissions is 0.6%. There are only three explanations. Either the tools are not actually used in submission grade work, which the industry evidence makes implausible, or they are used and not disclosed, which the survey evidence makes likely. Alternatively, the disclosure requirement is understood to apply to authorship rather than to method, so a systematic review screened by a large language model is not thought to require declaration in the way a manuscript drafted by one would be. The third explanation carries most of the weight and is fixable, because it is a definitional failure rather than a compliance failure. Current instruments ask whether AI wrote the text. Reviewers need to know whether AI selected the evidence, defined the cohort, extracted the parameter, or generated the code.

### What Acemoglu’s Critique Means for This Field

Daron Acemoglu, awarded the 2024 Sveriges Riksbank Prize in Economic Sciences in Memory of Alfred Nobel with Simon Johnson and James Robinson,^132^ estimates total factor productivity gains no greater than 0.66% over 10 years, roughly 0.064% annually, with gross domestic product effects near 1%. The estimate derives from a task-based decomposition rather than from pessimism about model capability.^133,134^ His central mechanism is the distinction between easy and hard learning tasks. A task is easy to learn when outcomes are observed, labels are abundant, and feedback is fast, and hard to learn when the correct answer is not observed. His illustration is clinical: diagnosing a persistent cough is hard because “there is no large, well-curated data set of successful diagnoses and cures.”

Health economics is the limiting case of a hard learning task, for structural rather than technological reasons. The quantity of interest is a counterfactual, so no data set of correct incremental cost effectiveness ratios exists, will exist, or could exist. This is why the empirical pattern reported in Part 1 is exactly what his framework predicts: the gains disappear precisely where the label disappears.

Acemoglu’s second mechanism is task share. Even large improvements on a small share of total task time yield small aggregate gains.^135,136^ Applied to HEOR, the clerical layer is where the 40% to 97% figures live, and it is a minority of the total work and the least remunerated portion of it. The judgment layer, choosing the comparator, specifying the model structure, defending the extrapolation, negotiating the price, is unaffected. Under this arithmetic an 80% reduction in a task that is 20% of the total yields a 16% aggregate gain in the best case, before accounting for the review burden the automation creates.

The counterargument deserves fair statement, because it is empirically supported. Noy and Zhang found writing time down 40% and quality up 18% across 453 professionals with the largest gains for weaker performers,^137^ Brynjolfsson et al found 14% to 15% more resolutions per hour across 5179 support agents with 34% gains for novices,^138,139^ and Peng et al found programmers 55.8% faster.^140^ Three features are uncomfortable for HEOR. The gains concentrate in the least experienced, which compresses the junior training pathway through which HEOR expertise has been built. They are largest where output is verifiable, which HEOR output is not. And the direction reverses outside the frontier of capability: in a randomized study of 758 consultants, those using AI inside that frontier completed 12.2% more tasks at a 25.1% faster rate, while on a task outside it, accuracy fell from 84.5% to 70.6% with AI and to 60.0% with AI plus an overview of its limits.^141^ The overview made it worse. HEOR’s hardest tasks sit outside the frontier, and its practitioners cannot yet reliably tell which side a given task is on, since 61% rate their proficiency as basic or very limited.^117^

What follows is not that AI will not matter. It is that the mechanism by which it matters will be cost structure rather than evidence quality, and that the two are separable. The realistic 10-year expectation is a HEOR function producing more dossiers per analyst at similar quality, with a compressed junior tier, a widened tail of low-quality submissions from parties who could not previously afford to submit at all, and a corresponding increase in the burden on reviewers. Reviewers, not producers, absorb the externality. That is the case for the reporting standard below, and it is an economic case rather than an ethical one.

### TRACE-HEOR: A Reporting Standard for AI-Assisted HEOR Evidence

Existing instruments cover the parts of this problem that resemble clinical prediction or clinical trials: TRIPOD+AI and PROBAST+AI for prediction model reporting and bias,^142,143^ CONSORT-AI and SPIRIT-AI for trials of AI interventions,^144,145^ DECIDE-AI for early live clinical evaluation,^146^ STARD-AI for diagnostic accuracy,^147^ TRIPOD-LLM for large language model studies,^148^ CANGARU for disclosure in scholarly writing,^149^ CHEERS 2022 for economic evaluation reporting,^150^ PALISADE for real-world data study credibility,^151^ and ELEVATE-GenAI with the ISPOR taxonomy for the HEOR-specific work.^152–154^ None answers the question an HTA reviewer actually has, which is whether the reimbursement recommendation would change if the AI component were removed.

We propose TRACE-HEOR, for Transparent Reporting of Artificial intelligence Components in Health Economics and Outcomes Research. The following 10 items are designed as an extension to CHEERS 2022 and PALISADE rather than as a replacement, and stated so that each is verifiable by a reviewer with access only to the submission. **Table 3** presents them in full, with the evidence supporting each.

**Table 3.** TRACE-HEOR: Proposed 10-Item Standard for Transparent Reporting of AI Components in Health Economics and Outcomes Research

**Item**	**Short Name**	**Requirement**	**Empirical Basis**	**Reviewer Verifiable**
1	Function, not tool	Declare, for each AI component, which function it performed: evidence identification or screening, data extraction, cohort or exposure definition, outcome or endpoint derivation, covariate or confounder selection, effect estimation, parameter derivation, model code generation, synthetic data or external control generation, or narrative drafting. Name the model, version, vendor, and dates of use.	Disclosure instruments currently ask whether AI wrote the text. Disclosed AI use was 3/1391 HTA documents (0.2%) and 0.6% of *Lancet* submissions, against self-reported use of 25%-69%.	Yes
2	Context of use and model risk	State the question of interest, the context of use, and the model risk using the 2-factor definition of model influence and decision consequence; name the decision the model influences.	Adopts the FDA credibility assessment framework verbatim so that a single characterization serves both regulatory and HTA review.	Yes
3	Calibration, not only discrimination	Report calibration in the deployment population and time period, not only discrimination. Where the estimate depends on absolute risk or absolute cost, discrimination alone is insufficient.	Across 4 deployed clinical AI systems, calibration degraded earlier and more consistently than discrimination. Cost effectiveness results depend on calibration.	Yes
4	Reliability of the human reference	Where AI output is validated against human abstraction, report the reliability of that human reference, including inter-abstractor agreement.	The reference standard for scaled machine learning response abstraction itself had agreement of 0.94 on assessment presence and 0.82 on best response category.	Yes
5	Disaggregated, variable level performance	Report performance separately for each variable the analysis depends on. Aggregate F1 or accuracy is not sufficient.	Stage value reached 98.8% precision while histology linkage reached 79% recall in the same model; T stage reached F1 0.89 while M stage reached 0.24.	Yes
6	Decision reversal analysis	Re-estimate the primary result with the AI component replaced by the pre-AI alternative or by a plausible worst case, and state whether the incremental cost effectiveness ratio crosses the relevant threshold or whether the recommendation changes.	No existing instrument requires this. Deriving real-world progression from radiology reports rather than clinical notes moved median survival by 7 months, which is decision relevant.	Yes
7	Bias and subgroup performance	Report performance for the subgroups the decision affects and report the demographic composition of any training or synthetic population.	A commercial risk algorithm applied to approximately 200 million people identified 17.7% of Black patients for extra help where a corrected target identified 46.5%.	Yes
8	Synthetic data and external controls	Report generator family, fidelity against the real target quantity rather than against summary statistics, and a privacy evaluation.	Generator choice alone moved recovery of the true median progression free survival from 6.5% to 98.1% of replicates. Only 11% of studies used a differentially private generator.	Yes
9	Human oversight actually performed	Report quantitatively what fraction of AI output was reviewed, by whom, with what qualifications, and what the disagreement rate was.	A biased AI lowered clinician diagnostic accuracy by 11.3 percentage points and explanations did not protect; experienced radiologists fell from 82.3% to 45.5% with an incorrect suggestion.	Yes
10	Archive prompts, code, and versions	Deposit prompts, code, model versions, random seeds, and intermediate outputs in a citable archive at the time of submission.	5% of health-economic evaluations mention code availability. An AI assisted analysis is not auditable without the prompts.	Yes

TRACE-HEOR is designed as an extension to CHEERS 2022 and PALISADE rather than as a replacement and is aligned to the FDA credibility assessment framework and to ELEVATE-GenAI. Items 1, 2, 6, 9, and 10 constitute the minimum set. Item 6 is the item no current instrument requires and the item a reimbursement committee needs most. Every item is stated so that it can be verified by a reviewer with access only to the submission.

1. Declare the function, not the tool. State, for each AI component, which of the following it performed: evidence identification or screening, data extraction, cohort or exposure definition, outcome or endpoint derivation, covariate selection, effect estimation, parameter derivation, model code generation, synthetic data or external control generation, or narrative drafting. Name the model, version, vendor, and dates of use, as *JAMA* already requires.^104^
2. Specify the context of use and the model risk, using the FDA’s 2-factor definition of model influence and decision consequence,^5^ and state which decision the model influences.
3. Report calibration, not only discrimination, in the deployment population and time period. Where an estimate depends on absolute risk or absolute cost, discrimination alone is insufficient.^69^
4. Report the human reference standard and its own reliability. Where AI output is validated against human abstraction, report inter-abstractor agreement for the reference.^155,156^
5. Report performance for the variables the analysis depends on, disaggregated. Aggregate F1 conceals the pattern in which stage extraction reaches 0.988 precision, while histology linkage reaches 0.79 recall^157^ and T stage reaches F1 0.89 while M stage reaches 0.24.^158^
6. Report a decision reversal analysis. Re-estimate the primary result with the AI component replaced by the pre-AI alternative, or by a plausible worst case, and state whether the incremental cost-effectiveness ratio crosses the relevant threshold or whether the recommendation changes. This item exists in no current instrument and is the one reviewers need most.
7. Report bias and subgroup performance for the populations the decision affects and state the demographic composition of any training or synthetic population.^64,159^
8. For synthetic data or model based external controls, report generator family, fidelity against the real target quantity rather than against summary statistics, and a privacy evaluation. Generator choice alone moved recovery of the true median progression free survival from 6.5% to 98.1% of replicates.^160,161^
9. Report the human oversight actually performed, quantitatively: what fraction of AI output was reviewed, by whom, with what qualifications, and the disagreement rate.^70,71,76,119^
10. Deposit prompts, code, model versions, seeds, and intermediate outputs in a citable archive at submission. Five percent of health economic evaluations currently mention code availability,^93^ and an AI-assisted analysis is not auditable without the prompts.

Items 1, 2, 6, 9, and 10 are the TRACE-HEOR minimum set, and a submission reporting only those five is materially more auditable than any submission we located. If an agency were to adopt only one, it should be item 6, because decision reversal is the only item that converts a technical property of a model into the currency a reimbursement committee reasons in.

### A Research Agenda

Five priorities follow, ordered by the ratio of decision relevance to current investment.

First, benchmark AI-derived endpoints against regulatory grade human abstraction in a multisponsor, multivendor study with a preregistered protocol, and publish the discordant cases. Current validation is vendor-authored,^156,162,163^ which is a conflict rather than a failing, and the remedy is independent replication.

Second, obtain a regulatory precedent. Sponsors should seek scientific advice or a qualification opinion on an AI-derived endpoint, and agencies should publish the reasoning when they give it, as the EMA did on prognostic covariate adjustment.^92^ One published opinion would be worth more to this field than 50 conference abstracts.

Third, establish an empirical decision reversal literature. Take completed cost-effectiveness analyses and HTA submissions, substitute AI-assisted components for the original ones, and measure how often the recommendation changes. The replication literature indicates the base rate of nonreplication is already high before AI is introduced,^98,99^ which makes the marginal question both answerable and urgent.

Fourth, run randomized trials of AI-assisted HEOR workflows with quality, not speed, as the primary endpoint. Every existing industry efficiency figure is single-arm and self-reported, and the designs already exist in adjacent fields.^2,137,141^

Fifth, evaluate AI itself economically at a scale proportionate to its deployment, and build HEOR-specific competency and audit capacity. Nineteen published economic evaluations against 1614 authorized devices is a ratio of about 1:85^9,83^; the finding that no price per scan made an AI cost-effective in a national screening program should be reproduced or refuted,^86,87^ and reviewer side capacity is the binding constraint because joint clinical assessment volume rises on a fixed schedule.^28,32,117^

### Limitations

The regulatory, HTA, and industry record assembled here is a narrative review rather than a systematic one, and negative results are less visible in it: a sponsor that abandoned an AI-assisted approach has no reason to publish that fact. Several central quantities rest on conference abstracts and company communications, which we label rather than exclude because they are the only available source. The disclosure denominators are audits of published documents, so they measure disclosure and not use. TRACE-HEOR is a proposal derived from documented failure modes rather than a consensus instrument; it has not been through a Delphi process, and it should be treated as a starting draft for one.[Bibr ref-640173]

## CONCLUSIONS

Regulators have written more about AI in evidence generation than sponsors have disclosed using it. The FDA has authorized 1614 AI-enabled devices and received more than 500 drug and biologic submissions containing AI components, while its credibility framework remains a draft that excludes discovery and operational uses. Industry reports time savings of 40% to 97% in regulatory writing, none independently replicated. Three of 1391 health technology assessment documents disclose an AI component.

The gap is not primarily a compliance problem. Disclosure instruments were built to establish who wrote a document, and the operations that matter in this field are the ones that select the evidence, define the cohort, derive the endpoint, and generate the code. Under the task-based productivity arithmetic that Acemoglu applies to the wider economy, the tasks AI does well in health economics are the minority of the work and the least remunerated part of it, while the burden of checking the output falls on reviewers who did not choose the tool. TRACE-HEOR states ten items that would make an AI-assisted submission auditable, and one of them, a decision reversal analysis, would tell a reimbursement committee the only thing it needs to know.

### Disclosures

The author reports no conflicts of interest related to this work. No funding was received for this study. The author is affiliated with an academic institution and a health economics consultancy that conducts real-world evidence studies for pharmaceutical and life sciences clients; no client had any role in the conception, conduct, analysis, or reporting of this review, and no client reviewed the manuscript before submission. The large language model classifier described in Part 1 is a measured object of study in this series rather than an assistive writing tool. Its prompts, inputs, and outputs are archived in the replication package. No AI tool is listed as an author.

## Supporting information

Online Supplementary Material

## Data Availability

All search strings, retrieved records, the classification dictionary, the blinded classifier prompts and outputs, the analysis scripts, the figure generation code, the regulatory and industry source tables, and the complete reference library are available in the public replication package 4. No individual patient data were used.
