# Supplementary material for: Faster but Not Truer, Part 2: The Regulatory Record, the Economics, and the TRACE-HEOR Reporting Standard
Source: J Health Econ Outcomes Res. 2026 Oct 15;13(2):161–77. doi: 10.36469/001c.171942 (PMC13676314; doi:10.36469/001c.171942)
Supplement: Supplementary file 1 [file jheor_2026_13_2_171942_371410.pdf]

Supplementary Appendix

Faster but Not Truer, Part 2: The Regulatory Record, the Economics, and TRACE-HEOR. JHEOR. 2026;13(2):161-177. doi:10.36469/jheor.2026.171942

Table S1 through Table S5 support the Results of Part 2. Provenance labels are Peer reviewed, Preprint, Conference abstract, Company communication, and Not available. The replication package is at https://github.com/obaser3791/ai-heor-scoping-review.

Table S1. Published economic evaluations of AI in health care

Study or intervention	Setting	Result	Interpretation
Systematic review of economic evaluations of AI	2020 to 2024	19 economic evaluations identified; CHEERS scores 21 to 27 of 28	One published economic evaluation for approximately every 85 authorized devices
AI versus polygenic risk scores for breast cancer risk stratification	United States	ICER US$23,755 per QALY in 2020 US dollars; AI cost threshold US$318 per mammogram	Cost effective within conventional thresholds under the modelled assumptions
AI intervention, dual perspective analysis	Not stated	US$1,107.63 per QALY health system perspective; US$10,347.12 per QALY societal perspective	Perspective choice moved the ratio by roughly an order of magnitude
AI in the UK national breast screening programme	United Kingdom	A scenario was identified in which no price per scan made the technology cost effective	Cost effectiveness was determined by the downstream care pathway rather than by algorithm price
Risk based breast cancer screening model	United States	Deep learning risk model enabling risk stratified screening intervals	Provides the clinical basis that the economic evaluations above assess
Framework for valuing clinical decision support	Methodological	Sets out how decision support value accrues through changes in downstream care	Explains the mechanism behind the UK screening result
Reinforcement learning for tuberculosis screening	Not stated	Illustrates the sequential decision variant of the evaluation problem	Extends the framework to adaptive interventions

A systematic review identified 19 economic evaluations of AI in health care published between 2020 and 2024, with CHEERS scores between 21 and 27 of 28, against 1,614 FDA authorized AI enabled devices. The examples below illustrate why the price of the algorithm is frequently the wrong parameter to negotiate.

Table S2. FDA authorized AI-enabled medical devices by review panel and year of final decision

Review panel (lead)	Authorizations (n)	% of all	Before 2016	2016	2017	2018	2019	2020	2021	2022	2023	2024	2025	2026
Radiology	1,230	76.2	15	11	16	40	51	99	110	138	178	183	254	135
Cardiovascular	154	9.5	4	4	7	10	15	8	11	14	21	15	30	15
Neurology	73	4.5	2	1	0	6	5	1	3	2	11	15	19	8
Anesthesiology	30	1.9	3	0	1	0	1	1	0	2	5	5	8	4
Gastroenterology-Urology	27	1.7	0	0	0	1	0	0	3	1	5	5	8	4
Hematology	22	1.4	6	0	1	2	1	3	0	2	1	1	3	2
Pathology	10	0.6	2	0	0	0	0	0	1	0	1	1	4	1
Ophthalmic	10	0.6	0	0	0	1	1	1	1	2	1	2	1	0
General and Plastic Surgery	9	0.6	2	0	1	0	0	0	0	0	0	2	2	2
Clinical Chemistry	9	0.6	1	1	0	2	2	0	0	1	2	0	0	0
Orthopedic	8	0.5	0	0	0	0	0	0	0	0	1	1	3	3
Dental	7	0.4	0	0	0	2	0	0	0	0	0	3	0	2
Microbiology	7	0.4	2	0	0	0	2	1	0	0	0	0	1	1
General Hospital	6	0.4	0	1	0	0	1	0	0	0	0	2	0	2
Obstetrics and Gynecology	5	0.3	1	0	0	0	1	0	0	0	0	0	2	1
Clinical Toxcicology	5	0.3	4	0	0	1	0	0	0	0	0	0	0	0
Ear Nose & Throat	1	0.1	0	0	0	0	0	0	0	0	0	0	0	1
Immunology	1	0.1	0	0	1	0	0	0	0	0	0	0	0	0
All panels	1,614	100.0	42	18	27	65	80	114	129	162	226	235	335	181

Retrieved from the FDA AI-Enabled Medical Device List, current as of 4 September 2026, and tabulated in full: 1,614 authorizations. The 2026 count covers decisions through the retrieval date only. Three quarters of authorized AI in United States medicine operates in a single specialty, which bounds how far the cost effectiveness literature reviewed in Table S1 can generalize. Decisions before 2016 are collapsed into a single column.

Table S3. Industry reported AI deployment in pharmaceutical evidence generation, with provenance

Organization or partnership	Application	Reported figure	Baseline or comparator	Provenance
Pfizer	Clinical study report and manuscript authoring	First draft CSR 40% faster; manuscript submission timeline 15% shorter; enrollment target plus 20%	Historical internal process	Company communication
Merck	Clinical study report authoring	180 hours to 80 hours per fully human reviewed first draft; errors reduced 50%; first draft in as little as five minutes; team of more than 80 across three continents	Historical internal process	Company communication
Merck and Google Cloud	Enterprise AI platform	Agreement valued at up to US$1 billion over multiple years, announced 22 April 2026, covering 75,000 employees	Not applicable	Company communication
Novo Nordisk, NovoScribe	Clinical study documentation	More than ten weeks to ten minutes; 95% reduction for device verification protocols; CSR writing time cut 90%; review cycles halved; baseline 2.3 CSRs per writer per year with a team of 11	Historical internal process	Company communication
Takeda	Analysis and tabulation, document summarisation	A 20 week process projected to be cut approximately in half; 70 pages summarised in two sentences	Historical internal process	Company communication
Takeda and Weave Bio	IND document generation	Approximately 100 hours to 3.7 hours for 18,870 pages across 61 documents in IND-1; to 2.6 hours for 11,425 pages across 58 documents in IND-2	Historical estimate, no concurrent control, n = 2 INDs, vendor co-authored	Preprint
Certara, CoAuthor	Regulatory writing	Baseline of approximately 120 hours over four weeks and 83 days from database lock to approved CSR; no time saving reported for the product itself	Not applicable, baseline only	Company communication
Novartis	Site selection for a 14,000 patient cardiovascular outcomes trial	Four to six weeks reduced to a two hour meeting; enrolment closed 13 patients above target	Historical internal process	Company communication
Novartis	Responsible AI programme	More than 100 use cases; adverse events reported within 24 hours; targets of two years faster development and US$1 billion to US$2 billion cost reduction	Historical internal process	Company communication

Organization or partnership	Application	Reported figure	Baseline or comparator	Provenance
GSK	Late stage study efficiency	Approximately GBP 8 million (US$10.87 million) saved in late stage asthma studies; all trials targeted 15% faster	Historical internal process	Company communication
Eli Lilly and NVIDIA	Compute infrastructure	More than 1,000 B300 graphics processing units, announced 28 October 2025	Not applicable	Company communication
Tempus, AstraZeneca, and Pathos	Multimodal oncology foundation model	US$200 million in data licensing and model development fees, announced 23 April 2025	Not applicable	Company communication
Moderna and OpenAI	Enterprise generative AI adoption	750 custom assistants deployed in two months; internal assistant adoption above 80%	Not applicable	Company communication
Pharmacoevidence	AMCP Format v5.0 payer dossier drafting	Draft approximately 90% complete; approximately 80% less development time; 70% to 75% lower cost; authors noted repetition in all sections and only somewhat complete SME agreement	Single team, no comparator, 110 source documents	Conference abstract
Norstella	Payer policy and formulary monitoring	Alerts within 24 to 48 hours; analysis in minutes rather than days or weeks	Not stated	Company communication
IQVIA	Market access analytics	No quantitative figure reported on the product materials	Not applicable	Not available
Exscientia and Sumitomo, DSP-1181	AI designed candidate	Entered the clinic January 2020, abandoned January 2022 for failing to meet study criteria	Not applicable	Peer reviewed
Exscientia, EXS-21546	AI designed candidate	Discontinued after modelling suggested a suitable therapeutic index would be difficult to reach	Not applicable	Peer reviewed
Exscientia, BenevolentAI, and Recursion	AI discovery platforms	Combined accumulated deficit above US$1.5 billion; equity values down at least 75%	Not applicable	Peer reviewed
Recursion, REC-994	Phase 2 in cerebral cavernous malformation	62 patients; trend toward reduced lesion volume on imaging but no improvement on patient reported outcomes or physician led measures at 12 months	Placebo controlled	Company communication

Organization or partnership	Application	Reported figure	Baseline or comparator	Provenance
AI discovered molecules, independent analysis	Phase transition success	Phase 1 success 80% to 90%; phase 2 success approximately 40%	Historical 13.8% phase 1 to approval overall; 3.4% in oncology	Peer reviewed
AI enabled assets, industry wide analysis	Clinical pipeline	117 assets across 63 companies; 60 of 117 (51.3%) completed phase 1; 8 of 117 (6.8%) completed phase 2	Total clinical pipeline of several thousand	Peer reviewed

Every efficiency figure in this table is reported by the organization deploying or selling the tool. None has been independently replicated, and none is accompanied by a concurrent control. The most methodologically explicit, the IND document generation preprint, compares against a historical estimate with n = 2. Provenance labels are as in Table 4.

Table S4. Journal, publisher, and society policies on the disclosure of AI use

Body	Requirement	Notable provision
ICMJE	Disclosure at submission; AI cannot be an author	Authors are accountable for all content, including AI generated portions
COPE	AI tools cannot be listed as authors	Authors must be able to assert the presence or absence of AI generated content
NEJM AI	Disclosure of authoring tools; reviewer disclosure	Reviewers must disclose AI use and protect manuscript confidentiality
JAMA Network	Disclosure of model name, version, manufacturer, and dates of use	The most specific technical disclosure requirement among general medical journals
Health Affairs	Disclosure in the cover letter and in Methods or Acknowledgements	Requires disclosure in the manuscript body, not only to editors
The Lancet	Disclosure required; AI peer review prohibited	States that use of AI for peer reviewing is not currently permitted
Elsevier	Named declaration section required	Separates permitted language improvement from reportable analytic use
Springer Nature	Documentation in Methods; copy editing exempt	Large language models do not satisfy authorship criteria
ISPOR and Value in Health	Disclosure of AI generated content; AI may not be cited as a reference	States that authors may not attempt to hide or disguise their use of AI

Observed disclosure runs far below reported use. The Lancet editors checked every submission received in February 2024 and found 0.6% declaring ChatGPT, 0.6% Grammarly, and 1.8% AI as a research tool, against 3.6% reporting a medical writer. The common structural gap is that these instruments govern authorship of text rather than the analytic functions that move an estimate.

Table S5. Adoption, workforce, and transparency surveys bearing on AI in HEOR

Source and population	Field date	n	Key finding
HEOR leaders at 72 companies	2025 to 2026	100 leaders, 58 to 59 AI items	69% personally use AI; 80% say the employer supports it; 29% expect AI to replace HEOR staff within two years against 47% who disagree; 59% report the team does not use AI for any listed workflow
HEOR, real-world data, and medical affairs professionals	April 2026	133	Expert 4%, advanced 11%, moderate 24%, basic 52%, very limited 9%, so 61% basic or very limited
Physicians, AMA augmented intelligence survey	2023, 2024, 2026	1,081; 1,183; 1,692	38% using in 2023; 66% in 2024; 81% aware or using in 2026, with average use cases rising from 1.1 to 2.3; 88% concerned about skill loss; 88% want safety and efficacy validation
US healthcare leaders, generative AI implementation	Q4 2023 to Q4 2025	150	25% to 47% to 50% implemented; agentic AI implemented 19% with 51% in proof of concept; 82% expect positive return but only 45% have quantified it
Medical affairs professionals	2026	Not stated	86% confident AI improves efficiency; leading edge 20%, cautious 41%, fast followers 33%, wait and see 6%; top barrier validation and accuracy 47%; 49% use full manual SME review of every AI generated piece
Life sciences generative AI budgets	2024 to 2025	82	Budgets of at least US$5 million rose from 20% to 32%
Life sciences executives	January 2026	200	74% rate AI crucial or very important; 60% expect budget increases overall and 71% in human pharma; 82% concerned about intellectual property liability
ISPOR and ISPE conference abstracts	1 January 2020 to 6 December 2024	28,184 abstracts	1,153 AI related; 89% of applications in systematic literature review automation and 9% in economic modeling; CRO 44%, academia 40%, pharmaceutical 16%
HTA agency practice and submissions	2023 to 2025	1,309 screened, 37 included	Seven agencies adopting AI internally; NICE recorded 49 AI initiatives across 25 HTAs
HTA dossier disclosure audit	2023 to 2025	1,391 documents	3 documents (0.2%) disclosed AI or machine learning, all at NICE (TA1154, TA1071, TA962); no explicit reporting in CDA or IQWiG dossiers
Journal submission disclosure audit	February 2024	All submissions in one month	0.6% declared ChatGPT, 0.6% Grammarly, 1.8% AI as a research tool, against 3.6% reporting a medical writer
Researchers, Nature survey	2025	5,229	Divided on acceptability, with roughly a quarter reporting use of AI to edit papers
Academics on AI in peer review	2025	Approximately 1,600 across 111 countries	More than half reported having used AI in peer review

Source and population	Field date	n	Key finding
Health economic evaluation transparency	2019 sample	200 evaluations in 147 journals	10 (5%) mentioned access to raw data and analytic code; 112 (56%) reported data to recreate the base case ICER; 31 (16%) reported working from a protocol
Discrete event simulation model sharing	2019 to 2022	564 studies	47 (8.3%) cited an openly available model, rising from 4.0% to 9.0%
Open source health economic models	Not stated	8,664 screened, 182 models	74% hosted on GitHub, 64% written in R, 49% Markov structure
US digital health venture funding	2019 to 2025	482 deals in 2025	AI enabled companies took 37% of funding across 191 deals in 2024, rising to 54% of funding and 50% of deals in 2025, with a deal size premium of approximately 19%

Self reported use ranges from 25% to 81% depending on population and year, against disclosed use of 0.2% in HTA documents and 0.6% in journal submissions. Within HEOR specifically, proficiency is low and team level adoption lags individual use.
